# Draft genome of a deep-sea *Bacillus* from sediments in the Alarcón Rise vent field

**DOI:** 10.1128/mra.00158-26

**Published:** 2026-07-20

**Authors:** Abril Hernandez-Monroy, Jaime Gasca-Pineda, Marcos Paolinelli, Tzvetanka D. Dinkova, Píndaro Díaz Jaimes, Patricia Velez

**Affiliations:** 1 Departamento de Botánica, Instituto de Biología, Universidad Nacional Autónoma de México 7180, Mexico City, Mexico; 2 Posgrado en Ciencias del Mar y Limnología, Universidad Nacional Autónoma de México 7180, Mexico City, Mexico; 3 Departamento de Biología Evolutiva, Instituto de Ecología, Universidad Nacional Autónoma de México 7180, Mexico City, Mexico; 4 Estación Experimental Agropecuaria Mendoza, Instituto Nacional de Tecnología Agropecuaria 42651, Luján de Cuyo, Mendoza, Argentina; 5 Consejo Nacional de Investigaciones Científicas y Técnicas (CONICET), CCT-Mendoza 63019 , Mendoza, Argentina; 6 Facultad de Química, Universidad Nacional Autónoma de México 7180, Mexico City, Mexico; 7 Instituto de Ciencias del Mar y Limnología, Universidad Nacional Autónoma de México 7180, Mexico City, Mexico; California State University San Marcos, San Marcos, California, USA

**Keywords:** secondary metabolism, microbial interactions

## Abstract

We report a draft genome of *Bacillus* sp. isolated from deep-sea sediment from hydrothermal vents. The genome size is 4.20 Mbp with a %GC content of 43.41. Our results highlight genes related to biofilm, chemotaxis, antimicrobials, and multidrug resistance. The extensive secondary metabolite diversity supports the presence of microbial interactions.

## ANNOUNCEMENT

Deep-sea hydrothermal vents are extreme ecosystems that host diverse microbial communities. Within these communities, heterotrophic bacteria are widely distributed in the heterotrophic belt, where fluid temperature is 8°C–18°C (1). They are significant contributors to carbon turnover and participate in key biogeochemical cycles, including hydrogen, sulfur, and nitrogen (2). However, the functional genomic potential of most of these heterotrophs remains largely unexplored. Here we present the draft genome of a *Bacillus* sp. isolated from sediment collected in the Alarcón Rise hydrothermal vent field (23°21.17′ N, 108°33.57′ W; depth: 2,176 m.b.s.l., nearly neutral pH; 3), Southern Gulf of California. Sediment cores were collected onboard the R/V Western Flyer (Monterey Bay Aquarium Research Institute, 2015). Under sterile conditions, a sub-sample from the core base was obtained, sealed, and frozen until isolation. In the laboratory, the inner portion of each core (15 × 3 cm) was collected and used for microbial isolation using the soil plating method (4) on potato dextrose agar and corn meal agar (Becton Dickinson) incubated at ∼22°C in darkness.

Genomic DNA was extracted using the QIAGEN Blood and Tissue DNA Extraction Kit. Library construction and genome sequencing were performed employing the Illumina DNA Prep Kit and Illumina-MiSeq Paired-End (2 × 150 bp) Reagent Kit v3 (300-cycles). Trimming was performed using Trimmomatic v0.40 (5) and *de novo* genome assembly with SPAdes v4.0.0 (6). Assembly quality statistics were retrieved from QUAST v5.2.0 (7). Small and overrepresented contigs were filtered, and assembly errors were corrected using Pilon v1.24.0 (8). Assembly completeness was assessed with BUSCO v6.0.0 (9), and the percentage of reads mapped to the assembly was calculated with BWA v0.7.19 (10). Functional annotation was performed using Bakta v1.11.3, Database v6.0 (11), and BV-BRC v3.54.6a (12), while secondary metabolite identification was retrieved with AntiSMASH v8.0.4 (13). Genome-based classification using GTDB-Tk v2.7.2 (14) assigned the isolate to the genus *Bacillus*. The closest reference genome corresponded to *Bacillus subtilis* (ANI = 98.14% and AF = 0.916).

The draft genome consists of 13 contigs with 33.6× coverage, an N50 of 1.03 Mbp, and an L50 of 2. Genome length is 4.20 Mb with a GC content of 43.41%. CheckM completeness assay identified 100% complete genes. Additionally, coarse consistency (99.7), fine consistency (99), and CheckM contamination (0.1) evidenced a high-quality assembly. Bakta gene prediction identified 49 tRNAs, 1 tmRNA, 4 rRNAs, 29 ncRNAs, 66 ncRNA regions, 4,296 CDSs, 16 pseudogenes, and 151 hypotheticals. Secondary metabolites identified correspond to plipastatin, surfactin, bacillaene, bacillibactin, subtilosin-A, and bacilycin. Also, biofilm, chemotaxis, and multidrug resistance were found within annotated genes. Most of these secondary metabolites have been reported during antagonistic microbial interactions with fungi (15–18). These findings are consistent with prior reports documenting potential antagonistic fungal-bacterial interactions in hydrothermal vents (19). Moreover, they agree with the identification of antimicrobial compounds from fungi isolated from hydrothermal systems (20), including the Alarcón Rise (21). Further *in vitro* studies must explore the molecular basis of these interactions and their ecological implications in the ecosystem.

## Data Availability

This project has been deposited in GenBank under accession number PRJNA1419894, including SRA raw reads (SRX32087875) and draft genome assembly (JBVFMU000000000.1). Bioinformatic methods are available at https://doi.org/10.6084/m9.figshare.32039886. Taxonomic classification results and annotations generated by Bakta, BV-BRC, and antiSMASH are publicly available on Figshare (https://doi.org/10.6084/m9.figshare.31975077). The *Bacillus* sp. isolate is deposited in the culture collection of Laboratory C-202, Instituto de Biología, Universidad Nacional Autónoma de México, headed by Dr. Patricia Velez, and is fully available for research upon request.
